# A Randomized Controlled Trial: Comparison of 4% Articaine versus 0.5% Bupivacaine for Ambulatory Orthopedic Surgery under Supraclavicular Block

**DOI:** 10.1155/2020/2194873

**Published:** 2020-09-24

**Authors:** Simon H. Armanious, Gamal A. Abdelhameed

**Affiliations:** ^1^Department of Anesthesia, Faculty of Medicine, Ain Shams University, Cairo, Egypt; ^2^Ain Shams University, Chairman of Anesthesia Department, Cairo, Egypt

## Abstract

**Background:**

Articaine has been used in many dental and ophthalmic outpatient procedures. In the era of ultrasound-guided regional techniques, we searched for short and potent local anesthetic for patients undergoing ambulatory upper limb procedures. However, studies about articaine efficacy in brachial plexus block are limited. In this study, we compared its safety and efficacy against bupivacaine as a commonly used anesthetic agent for ultrasound-guided supraclavicular brachial plexus block.

**Methods:**

This randomized prospective study was performed at Ain Shams University Hospital from January to March 2020. A total of 117 patients aged 20 to 60 years, with the American Society of Anesthesiologists physical status I and II, were enrolled in the study. Patients were randomly allocated into two groups: in group A, patients received 30 ml articaine 2%, and in group B, patients received 30 ml of bupivacaine 0.5%. We measured motor and sensory block duration as a primary outcome. Other secondary outcomes such as onset of block, duration of analgesia, patient satisfaction, and time to home discharge readiness were also measured.

**Results:**

We analyzed data collected from 97 patients. The motor block duration was significantly shorter in group A (165.73 ± 20.33 min) than in group B (220.27 ± 37.73 min). The onset of motor block was faster in group A (8.73 ± 4.33 min), and the postoperative VAS score was lower in group B. Patients in group A achieved an earlier home discharge of 289.67 ± 2.73 min.

**Conclusion:**

Earlier resolution of articaine block makes it more favorable than bupivacaine for ambulatory surgery. This trial is registered with (NCT04189198).

## 1. Introduction

Revolution in the surgical plane, advances in anesthetic techniques, and availability of new drugs, over the last 40 years, have led to a large switch to day-case surgery throughout the world [1]. Both the economic value and improved patient satisfaction make the National Health Service (NHS) recommend that at least 75% of all surgical procedures should be performed as day cases [2]. Ultrasound-guided regional anesthesia plays a vital role in overcoming the limitations of general anesthesia, and supraclavicular brachial plexus block (SBPB) is used safely for both urgent and scheduled surgeries of the upper limb on a day-case basis [3].

Many hospitals are running out of capacity, so elective procedures such as joint replacement are postponed due to lack of beds when they are occupied by causality and inpatients. Currently, the potential is to liberate hospital beds by increasing the number of patients returned to the home on the same day of surgery [4]. An ideal local anesthetic (LA) drug for day-case patients should have faster sensory onset time and a differential offset, with an earlier offset of motor than sensory blockade, enabling them to move their arm while having continued analgesia, to achieve an early hospital discharge [5]. Regional anesthetic operating lists and the presence of a “block room” increase efficiency, reduce turnover time, and permit confirmation of sufficient nerve blockade before the surgical procedure commences [6].

Articaine 4% and bupivacaine 0.5% are amide-based LA drugs, and both are comparable in potency [7]; however, bupivacaine contains an aromatic ring that enhances its lipid solubility and potency, so bupivacaine was prepared in 0.5% solution while articaine was prepared in 4% solution. Articaine has an ester group (thiophene ring), and its ester group makes it inactive after ester hydrolysis, while its thiophene ring contains sulfur atoms, resulting in a very low immunological reaction of articaine and lower neurotoxic potential [8]. Articaine diffuses more rapidly through tissues and is metabolized by nonspecific esterases in both tissues and blood. Bupivacaine 0.5% is a well-established long-acting LA, but it has been associated with more cardiotoxicity, especially when used at high concentrations or accidentally administered intravascular [7].

Previous trials focused on comparing the effect of different LA drugs, such as lidocaine, ropivacaine, prilocaine, and bupivacaine for supraclavicular block, while others used different types of additives [9]. We searched for a potent short-acting LA. Therefore, this study was conducted to compare articaine 4% and bupivacaine 0.5% in ambulatory upper limb surgeries regarding the duration of sensory and motor block as a primary outcome, while onset time for block, duration of analgesia, patient satisfaction, and other possible complications were secondary outcomes.

## 2. Methods

This prospective, randomized, double-blind study was conducted at Ain Shams University Hospital from January to March 2020. After approval of the Research Ethics Committee of Ain Shams University (R-92) in October 2019, the study was registered at Clinical Trials.gov (NCT04189198). One hundred and seventeen adult patients aged 20–60 years planned for upper limb surgery below the midhumerus with an expected time of less than 90 min usually under tourniquet, both scheduled, e.g., ganglion excision, carpal tunnel, trigger finger release, etc., and causality patients, e.g., tendon repair, K wiring, plating of the fractured bone, cut wrist, wound suturing, etc., with isolated upper limb injury.

Patients excluded were those with allergies to local anesthetic, those with ASA III and IV, patients who were refused to participate, uncooperative patients, patients who had infection at the site of injection, patients with no telephone available, who are living alone, who have bleeding disorder, and patients on anticoagulant drugs. Preoperative investigation for all patients included complete hemogram, X-ray chest, serum creatinine, blood sugar, and electrocardiogram for those above forty.

All patients were reviewed by an anesthesiologist on the same day of surgery for any medical history, ensuring fasting for at least 6 hours. The anesthesia plan was discussed with the patient, and the patient signed a consent form. The expected motor block duration should be clarified, and the patient should be given written instruction as to their conduct until normal motor power and sensation return. One family member may attend and sit with the patient until the patient goes to the block room.

Inside the block room, an eighteen-gauge cannula was inserted in the nonoperative arm, and a 7 mL·kg^−1^ crystalloid infusion was infused slowly. All patients were premedicated with 0.03 mg·kg^−1^ midazolam IV, 25 *µ*g fentanyl, and 1 mg granisetron. Heart rate beat min^−1^ and peripheral oxygen saturation (SpO_2_) were monitored, and noninvasive arterial blood pressure (NIBP) was measured at 10 min intervals during the procedure. The baseline values were recorded. Nasal oxygen 2 L·min^−1^ was administered throughout the procedure.

**Figure 1:**
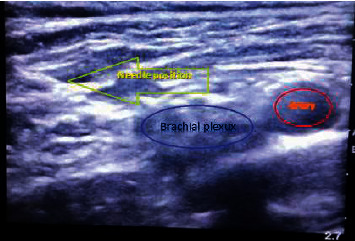
Needle position in the divisions of the brachial plexus seen as a bunch of grapes-like structures lateral and superficial to the subclavian artery.

**Figure 2:**
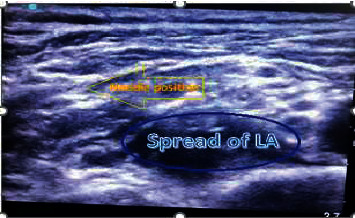
A spread of local anesthetic (LA) appears anechoic below the divisions of the brachial plexus with the needle in-plane from lateral to medial.

The patient was positioned in the supine position, his head was elevated 30° tilted to the contralateral side, and a transportable ultrasound system (SonoSite M-Turbo; SonoSite Inc., Bothell, WA, USA) with a 38 mm 8–13 MHz linear probe (HFL-38) was used to obtain clear images of the brachial plexus divisions and cords. An experienced anesthetist in nerve block techniques positioned the linear probe in the supraclavicular fossa. After aseptic skin preparation with povidone-iodine and LA infiltration by a blunt-tipped 25 G 3 ml syringe, the probe was covered by sterile gloves with water-based gel inside. An 11/4-inch 22 G spinal needle was advanced using an in-plane ultrasound technique. After careful aspiration, the local anesthetic solution was injected slowly in 5 ml increments (Figures 1 and 2).

In this randomized double-blind study, randomization was performed using computer-generated random numbers, and all patients were allocated to one of the two groups. The health care providing team (patients, anesthetists, surgeons, and nurses) was blinded to the patients' allocation. All information about patient allocation was kept by a pharmacist, who was not sharing in any further steps (data collection and analysis). All patients were assigned to receive a 30 ml volume of either articaine 2% in (group A) Ultracain® D articaine HCI 4% (no epinephrine) or bupivacaine 0.5% in (group B) bupivacaine HCL (Sunny pharmaceutical industries).

Sensory block and motor block were evaluated preoperatively to determine a baseline and every 5 min for 30 min or until onset of blockade was noted, which is earlier and thereafter every 60 min. Sensory block was assessed by the pinprick method (Grade (0) sharp pin felt, Grade (1) analgesia but dull sensation still felt, and Grade (2) complete anesthesia). Assessment of sensory block was done over the corresponding dermatomal distribution of the median nerve, radial nerve, ulnar nerve, and musculocutaneous nerve (thenar eminence, dorsum of the hand, hypothenar eminence, and lateral side of the forearm, respectively) until complete sensory blockade was attained. The onset of sensory block was considered at Grade (1) along with the distribution of any of the above nerve areas while Grade (2) refers to complete block [10].

Motor block was assessed by the modified Bromage scale [11] for the upper limb (Grade (0) patients were able to raise an extended arm, Grade (1) patients were weak but able to flex the elbow, Grade (2) patients were unable to flex the elbow, and Grade (3) patients had no movement in the whole limb). The onset of motor blockade was considered with Grade (1) Bromage, while Grade (3) refers to peak motor blockade. Patients were kept comfortable with arm by side, observed for signs of toxicity, and then transferred to the operating room, where hemodynamic parameters and vitals (blood pressure BP, heart rate HR, respiratory rate RR, and oxygen saturation SPO_2_) were monitored during the procedure. The block was considered to fail when sensory anesthesia was not achieved within 30 min. Patients who remained inpatient after the procedure or those with a failed block were excluded from the study.

After the end of the procedure, patients were transferred to a recovery area, drinks were served to them upon their request, oral medication could be resumed, the duration of sensory block was determined by noting the time when there was return of dull sensation to pinprick, and the duration of motor blockade was defined as the time interval between cessation of movement in the limb until Bromage (1). Both were recorded as a primary outcome. Other measured parameters including onset of sensory and motor block, patient satisfaction with the anesthetic technique using a special nurse record scale from 0 to 10 (where 0 = unsatisfied and 10 = fully satisfied), duration of analgesia using the visual analogue scale (VAS) measured postblock every 1 hour until VAS [5] (VAS score is a 10-point scale in which a score of “0” indicates “no pain” and a score of “10” indicates “worst pain imaginable”), and time to home readiness were all considered as a secondary outcome.

The postoperative analgesic strategy depends on the prescribed oral analgesic (1 g paracetamol/8 h), and rescue analgesia in the form of ketorolac (30 mg) was intramuscularly administered only at VAS [6]. The primary endpoint of the study was the time until complete recovery of the motor block Bromage scale (0), and patients met home discharge criteria (fully awake, 30>RR > 10, (SpO_2_) greater than 95% on room air, BP ± 20% of baseline, and written instruction was given to patients or companion) [12]. It is safe to discharge patients in arm sling with residual block if the patient requested that [13].

### 2.1. Statistical Method

A sample size of 44 patients per group was calculated to detect a 25% difference in the recovery time of motor power based on previous trials after articaine 2% injection in the brachial plexus was 172–185 min[14], while another study recorded that motor block duration of bupivacaine 0.5% was 216.27 ± 37.73 min [15], with a slandered deviation of 50 min using an alpha error of 0.05 and a beta error of 0.2 to compensate for patients who are excluded from during the study. We chose to randomize 50 patients in each group. Quantitative data are presented as mean ± SD, and an independent *t*-test or Mann–Whitney *U* test was used as a test of significance. Qualitative data were presented as frequencies and percentages; a chi-square test was used as a test of significance. A *P* value <0.05 was considered significant. The SPSS for Windows (version 10) statistical package (SPSS Inc., Chicago, IL) was used.

## 3. Results

**Figure 3:**
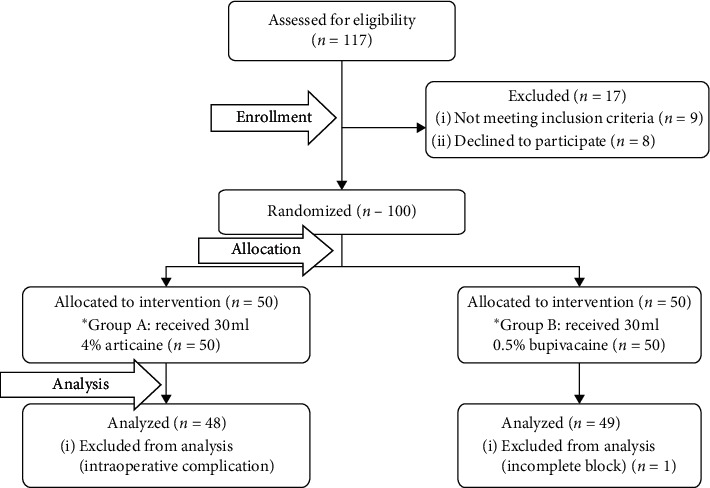
Consort diagram.

Of one hundred and seventeen patients invited to the study, seventeen patients were excluded from the study for different causes, 100 patients were randomized into 2 groups, two patients in the articaine group developed surgical bleeding with extended surgical time so excluded, and one patient in the bupivacaine group showed failed block and converted to general anesthesia. Finally, data were collected from 97 patients (Figure 3). The patient characteristics and type of surgery were comparable between both groups (Table 1). Six patients asked for additional intraoperative analgesia, and all were in the bupivacaine group and treated with 50 *µ*g fentanyl.

**Figure 4:**
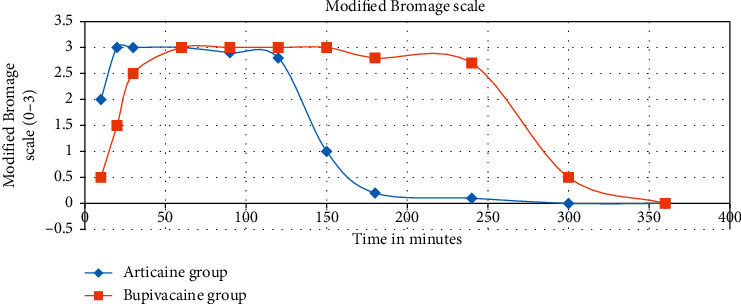
Modified Bromage scale showing early onset and offset of motor power in the articaine group.

There was a highly statistically significant difference in the time of onset of both sensory and motor block (*P* < 0.0001). The onset of motor block was faster in group A (8.73 ± 4.33 min) than in group B (17.53 ± 1.70 min). Regarding the duration of motor block (Figure 4), the obtained results showed highly significant prolongation in group B (220.27 ± 37.73 minutes), which was longer than that in group A (165.73 ± 20.33 minutes) (Table 2).

The VAS score in group A was lower than group B from the 1st hour of the study till the 3rd hour. After the 4th hour, patients in group B attain a higher VAS score than group A5). However, the time to reach a VAS score of 5 was 156.83 ± 30.96 min in group A and 166.33 ± 14.02 min in group B, which was statistically nonsignificant (Table 3). Although patients in group A were more satisfied with the anesthetic technique than those in group B, this was statistically nonsignificant. Patients in group A achieved earlier readiness to hospital discharge (289.67 ± 2.73 min) than those in group B (379.71 ± 30.27 min) (*P* < 0.0001), which was statistically highly significant.

## 4. Discussion

This study was conducted to compare the effect of 4% articaine with 0.5% bupivacaine for ambulatory upper limb short orthopedic procedures under supraclavicular block. The main finding was that ultrasound-guided brachial plexus block through a supraclavicular approach using 4% articaine can provide an effective surgical block of the upper limb while allowing an earlier home discharge from the hospital than that provided with 0.5% bupivacaine. Lidocaine, which was considered a gold slandered short-acting LA agent, has a very short duration of action and neurotoxic effect, which limits its use and forces investigators to use several additives such as adrenaline and dexamethasone to prolong the duration of block and decrease its neurotoxic effect [16]. Articaine has a shorter duration of action due to hydrolysis by nonspecific esterases in tissues and blood, leading to its rapid clearance [17].

Brachial plexus block is an ideal anesthetic choice for outpatient regional techniques; it provides surgical anesthesia for upper limb elective and emergency procedures with prolonged postoperative analgesia and does not interfere with ambulation [18]. The current study recorded a significantly faster regression of sensory and motor block in group A. Postoperative analgesia was significantly higher in the bupivacaine group, but patient satisfaction was comparable in both groups.

Motor block duration difference, the primary outcome of the current study, was significantly shorter by 75 min in the articaine group than in the bupivacaine group (Figure 4). Patients in the bupivacaine group experienced delayed return of motor function, which was reflected in the time of their hospital discharge. Five patients in the bupivacaine group were discharged to home after six hours with residual motor block with no recorded complications. The sensory block duration was also significantly shorter in the articaine group, as it is pharmacologically known that bupivacaine has a longer duration of action [8].

The local anesthetic doses used in the current study are clinically equivalent, as we used the minimum efficient dose without adding any additives to the LA drug injected, although articaine was studied before by Sert et al. [14] at a dose of 30 ml articaine 2% and motor block duration was 172–185 min, which is comparable to the current study motor block duration (165.73 ± 20.33 min). The main difference was in the targeted population and their patients were renal failure patients on regular hemodialysis, while we included healthy volunteers (ASA I and II).

In this study, bupivacaine was used in a volume of 30 ml at a 0.5% concentration, and several studies compared different bupivacaine volumes for many concentrations [19]. Gupta and Hopkins recommended that the effective dose 50 (ED_50_) for 0.5% bupivacaine was 26.8 ml because we did not use any additive to the LA drug. In our study, the mean duration of motor block in the bupivacaine group was 220.27 ± 37.73 min, and the difference in both groups was statistically significant with a *P* value < .0001. The motor block duration of bupivacaine was studied before by Rai and Kedareshvara [15], who tested the addition of dexamethasone to bupivacaine and observed that the mean duration of motor block of bupivacaine alone was 216.27 min. This result is similar to our study. Several additives to bupivacaine were studied before dexmedetomidine [20], fentanyl [21], nalbuphine [22], and magnesium [23]; all resulted in a significant prolongation of motor block duration.

In contrast to articaine, a significant delay in the onset of both sensory and motor block was recorded in the bupivacaine group with mean onset times of 8.73 ± 4.33 min and 17.53 ± 1.70 min, respectively, and the earlier onset of articaine action was related to its lower pKa (7.8) than that of bupivacaine (8.1). Normal tissue pKa is 7.4, which means that a greater portion of articaine will be present in the lipid-soluble form and ready to penetrate nerves [24]. The establishment of the peak effect of block in the articaine group was highly significant compared to the bupivacaine group (Figure 4) with a *P* value (*P* < 0.0001), which allowed earlier start of the surgical procedure and decreased overall hospital stay.

Although patient satisfaction was statistically nonsignificant between the two study groups, it was higher in the articaine group. This was attributed to earlier onset of the block and rapid resolution which allow early regain of full motor power and hospital discharge. A similar result was obtained in a study by Dijkstra et al. [25]. They compared hyperbaric articaine 80 mg versus hyperbaric bupivacaine 15 mg for spinal anesthesia in day-case surgery patients. They found an earlier resolution of spinal anesthesia with articaine by one hour, with a hospital discharge time of 300 min in the articaine group and 380 in the bupivacaine group, respectively.

**Figure 5:**
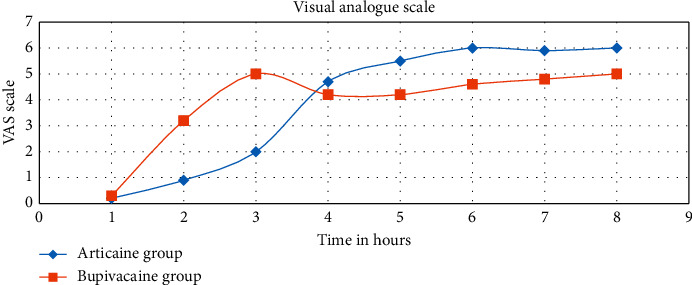
Visual analogue scale with low record in early hours and higher record postoperatively in the articaine group.

The visual analogue scale (VAS) was lower among patients in the articaine group during the first three hours (intraoperative time) compared with the bupivacaine group (Figure 5), so we found that the anesthetic efficiency of articaine was higher than that of bupivacaine, and none of the patients in the articaine group requested intraoperative analgesia, while six patients in the bupivacaine group asked for analgesia. This was attributed to higher diffusion of articaine in tissues and around nerve trunks, providing deep and solid anesthetic block [26]. Thakare and Kathariya also concluded that articaine was more efficient than bupivacaine during orthodontic extraction [7].

Patients in the articaine group experienced higher VAS scores in the postoperative time when compared with their bupivacaine counterparts. This may be explained by the higher protein binding capacity of bupivacaine and its higher pKa (8.1) [27]. Rapid metabolism of articaine (ester hydrolysis) explains its shorter postoperative analgesia duration. Although the patients in the bupivacaine group had prolonged postoperative analgesia, the prolonged motor block was unpleasant for them, and early regaining of motor function allowed patients in the articaine group to eat, drink, and avoid injury of the upper limb. This result was consistent with previous studies [28, 29].

In this study, an earlier ask for rescue analgesic medication in the articaine group was well managed by oral analgesics, and this does not delay hospital discharge. Patients in the articaine group discharged home much earlier, although other factors may influence hospital discharge such as availability of transportation, surgical discharge visit time, and patient motivation to go home. No patient in our study required hospital readmission after discharge.

## 5. Study Limitations

There is a lack of trials on articaine use in ultrasound-guided peripheral nerve blocks. Further studies with a lower volume of local anesthetics and with long-term follow up about the analgesic properties of both LA drugs will be needed.

## 6. Conclusion

We conclude that supraclavicular block with articaine results in a shorter duration of motor block than plain bupivacaine in upper limb orthopedic short procedures. Articaine is an efficient and safe alternative with a more accepted recovery profile for use in day-case surgery.

## Figures and Tables

**Table 1:** Patient demographics.

	Articaine group	Bupivacaine group	*P* value
Number of patients	48	49	—
Gender, *n* (%)	Female, 29 (60.4%)	Female, 21 (44%)	0.084
	Male, 19 (39.6%)	Male, 28 (56%)	—
BMI^##^	29.9 (3.1)	30.2 (3.3)	0.646
Age (±SD)	43.5 (±13.9)	43.2 (±14.7)	0.918
Elective surgery, *n* (%)	34 (70.8%)	28 (57.1%)	0.161
Emergency surgery, *n* (%)	14 (29.2%)	21 (42.9%)	—
Surgery duration, min (±SD)	26 (±15)	32 (±18)	0.078

Values are expressed as the mean (standard deviation), absolute number is expressed as (*n* (%)), *P* < 0.0001 to be statistically significant, and *P* > 0.05 to be nonsignificant.

**Table 2:** Block character.

	Articaine group	Bupivacaine group	*P* value
Duration of sensory block	216.75 ± 35.76	289.50 ± 45.71	<0.0001
Duration of motor block	165.73 ± 20.33	220.27 ± 37.73	<0.0001
Onset of sensory block	7.75 ± 3.76	12.43 ± 1.04	<0.0001
Onset of motor block	8.73 ± 4.33	17.53 ± 1.70	<0.0001

**Table 3:** Visual analogue scale and patient satisfaction.

	Articaine group	Bupivacaine group	*P* value
Time to VAS score of 5 in min	156.83 ± 30.96	166.33 ± 14.02	0.096
Patient satisfaction (/10)	9.2	9.1	0.59
Time to home discharge readiness in min	289.67 ± 2.73	379.71 ± 30.27	<0.0001

Data are expressed as the mean ± standard deviation, NS = *P* > 0.05 = not significant, and *S* = *P* < 0.0001 = significant.

## Data Availability

The data are available at https://www.synapse.org/#!Synapse:syn21817969/files/.Additionally, the data of this article are available from the corresponding author upon request via simondr106@gmail.com and simon_dr_106@hotmail.com
